# Stress Increases the Association between Cigarette Smoking and Mental Disorders, as Measured by the COVID-19-Related Worry Scale, in the Miami Adult Studies on HIV (MASH) Cohort during the Pandemic

**DOI:** 10.3390/ijerph19138207

**Published:** 2022-07-05

**Authors:** Janet Diaz-Martinez, Ivan Delgado-Enciso, Adriana Campa, Javier A. Tamargo, Haley R. Martin, Angelique Johnson, Suzanne Siminski, Pamina M. Gorbach, Marianna K. Baum

**Affiliations:** 1 Robert Stempel College of Public Health and Social Work, Florida International University, Miami, FL 33199, USA; ivan_delgado_enciso@ucol.mx (I.D.-E.); campaa@fiu.edu (A.C.); jtama025@fiu.edu (J.A.T.); halmarti@fiu.edu (H.R.M.); angjohns@fiu.edu (A.J.); 2 Faculty of Medicine, University of Colima, Colima 28040, Mexico; 3 Cancerology State Institute, Colima State Health Services, Colima 28040, Mexico; 4 Frontier Science Foundation, Brookline, MA 02446, USA; siminski@fstrf.org; 5 Fielding School of Public Health, University of California, Los Angeles, CA 90095, USA; pgorbach@ucla.edu

**Keywords:** smoking, COVID-19 stress, HIV

## Abstract

Background: Smoking has been associated with mental disorders (MD). People who smoke are at a higher risk of contracting COVID-19 and experiencing more severe symptoms of the illness. This study aimed to investigate the relationship between cigarette smoking and MD before and during the COVID-19 pandemic and whether it was influenced by COVID-19-related stress in the MASH cohort. Methods: An ambispective design was used with data collected during the pandemic (July/August 2020) by the COVID-19-Related Worry Scale, a parameter for stress, and data collected at the participants’ last cohort visit before the pandemic (December 2019). Results: In our sample of 314 participants, 58.6% were living with HIV, 39.2% had MD, 52.5% smoked before, and 47.8% smoked during the pandemic. Participants with MD were twice as likely to smoke cigarettes both before (aOR = 2.02, 95% CI: 1.21–3.37, *p* = 0.007) and during the pandemic (aOR = 2.10, 95% CI: 1.24–3.56, *p* = 0.006); and experienced higher levels of stress measured by the COVID-19-Related Worry Scale (8.59 [5.0–10.0] vs. 7.65 [5.0–10.0]; *p* = 0.026) compared to those without MD. Participants with MD and high levels of stress smoked more days per month (20.1 [0–30] days) than those with lower levels of stress (9.2 [0–30] days, *p* = 0.021), and more than those with high levels of stress, but no MD (2.6 [0–30] days, *p* < 0.001). Conclusions: Cigarette smoking decreased in the MASH cohort during the pandemic, but increased in participants with MD and higher levels of stress.

## 1. Introduction

Every year, smoking is responsible for over eight million deaths worldwide; seven million are directly related to cigarettes, and approximately 1.2 million are attributed to second-hand smoke [1]. In the United States (U.S.), cigarette smoking remains the leading cause of preventable disease, disability, and death [2]. A mental disorder, also referred to as a mental health condition, is defined by the World Health Organization (W.H.O.) and American Psychiatric Association (A.P.A.) as a disorder characterized by a clinically significant disturbance in an individual’s cognition, emotional regulation, or behavior [3,4]. There are many different types of mental disorders such as anxiety disorders, depression, bipolar disorder, schizophrenia, disruptive behavior disorders, and dissocial disorders, among others [4].

People with mental disorders smoke two to four times more than the general population [5]. This relationship represents a concern during the current COVID-19 pandemic as emerging evidence shows that people who smoke may be at higher risk of contracting and experiencing more severe symptoms of COVID-19 [6]. In the context of human immunodeficiency virus (HIV), smoking increases the risk of poor health outcomes [7]. During the COVID-19 pandemic, polysubstance use, including tobacco, among people living with HIV (PLWH), represents an additional concern due to its potential link to poor antiretroviral therapy (ART) adherence [8].

Stress and anxiety may increase smoking and relapse among former smokers, as smokers may self-medicate with tobacco to manage their psychological symptoms [9,10,11]. However, the pandemic may also serve as a motivator to reducing or quitting smoking due to a perceived threat of COVID infection [12,13]. Individuals with pre-existing (before the COVID-19 pandemic) mental disorders are reporting more adverse psychological effects during the pandemic [14,15,16]. Therefore, it is plausible that the COVID-19 pandemic affects smoking behaviors by exacerbating stress, which may be more pronounced among vulnerable and disadvantaged populations who already face a high prevalence of smoking-related morbidity and mortality. The effect of the pandemic on smoking behaviors in this population has crucial implications for public health strategies and allocation of resources. Understanding how smoking has changed during the pandemic due to exacerbation of psychological stress is critical for providing smoking cessation and mental health support. Nevertheless, the psychosocial impact of the COVID-19 pandemic, and whether it drives tobacco use changes in vulnerable groups has not yet been sufficiently explored.

The Miami Adult Studies on HIV (MASH) cohort participants are primarily low-income, predominantly Black and Hispanic adults, living with and without HIV, with an increased predisposition to substance use disorders and tobacco use [17,18]. Using data from the MASH cohort, we explored the relationship between mental disorder(s) and cigarette smoking, and how the presence of COVID-19-related worry, a measure of stress, may modify this relationship.

This study aimed to investigate the relationship between cigarette smoking and mental disorder(s) before and during the COVID-19 pandemic and whether it was influenced by COVID-19-related stress in the MASH cohort. We hypothesized that participants with mental disorder(s) would have a higher risk of smoking and that stress related to COVID-19 would exacerbate smoking behavior.

## 2. Materials and Methods

### 2.1. Study Design

The Collaborating Consortium of Cohorts Producing NIDA Opportunities (C3PNO), funded by the National Institute on Drug Abuse (NIDA), launched the C3PNO/COVID-19 survey in May 2020. This survey aimed to examine changes in mental health (resilience, anxiety, and COVID-19-related worry), substance use, and HIV prevention during the COVID-19 pandemic in participating C3PNO cohorts [19]. The C3PNO/COVID-19 survey has been used in several HIV cohort studies [20,21,22,23] and includes validated questionnaires available at https://www.c3pno.org (accessed on 20 June 2022).

A convenience sample of 400 MASH cohort participants were randomly selected to participate. From July to August of 2020, MASH cohort participants were contacted to complete a telephone survey. In total, 330 participants were successfully contacted, and all of those contacted agreed to participate. Sixteen participants were excluded from the analyses due to missing data.

This study used an ambispective design, including cross-sectional and longitudinal analyses using data collected before and during the pandemic to compare cigarette smoking behavior. Data before the pandemic were collected from participants’ last cohort visit (December 2019), which took place 6.8 ± 2.9 months before the C3PNO/COVID-19 survey was administered (July/August 2020). 

The Florida International University Institutional Review Board approved this study protocol (IRB-15-0004-AM43), and each participant provided verbal informed consent for participation in the study.

### 2.2. Study Population

The MASH cohort includes over 1000 people living with and without HIV. It is comprised of mostly Black non-Hispanic and Hispanic middle-aged individuals recruited from local clinics, mainly from the Borinquen Health Care Center, food banks, and shelters in low-income urban sectors of Miami, Florida. Participants in the MASH cohort are surveyed every six months for various social and clinical data, including new medical diagnoses, morbidity of chronic conditions, healthcare access and utilization, medication adherence, social determinants of health, and use of alcohol, tobacco, and illicit substances. Participation in the MASH cohort requires confirmation of HIV status (positive or negative) from medical records with participants’ signed release of medical information.

### 2.3. Baseline Characteristics

We used MASH cohort data for sociodemographic characteristics including age, sex, race/ethnicity, educational level, and income; and for comorbidities, including HIV status. 

### 2.4. Mental Disorders

Hereafter, we will use the term “mental disorder(s)” to refer to the group of participants who reported depression, anxiety disorder, bipolar disorder, and/or schizophrenia at baseline (last MASH cohort visit before the pandemic).

### 2.5. Substance Use

Participants reported the use of tobacco, cannabis, and cocaine in the last the 30 days prior to the survey. For example, participants were asked: “Have you smoked in the last month?”, “How many days did you smoke in the last 30 days?”, and “How many cigarettes did you smoke per day?” The Alcohol Use Disorder Identification Test-Consumption (AUDIT-C) questionnaire was used to assess alcohol consumption [20]. Scores of ≥4 for men and ≥3 for women are indicative of alcohol misuse.

### 2.6. COVID-19-Related Worry Scale

The COVID-19-Related Worry Scale is a surrogate for perceived stress related to the pandemic. It assesses COVID-19-related worry with a 1–10 numerical rating scale, determined by response to the following question: “On a scale of one to ten, how worried are you about the COVID-19 pandemic? One being not worried at all, and ten being extremely worried.”

### 2.7. Statistical Analysis

Descriptive statistics are reported using mean ± standard deviation (SD) for normally distributed variables or median (with first and third quartiles, Q1–Q3) as the dispersion indicator for variables with a non-normal distribution. Percentages are reported for categorical variables. Student’s *t*-test (or non-parametric Mann-Whitney U test) and Chi-square tests were performed to compare outcomes between groups. Within-group differences in smoking prevalence pre-pandemic vs. during the pandemic were tested with McNemar’s test. Statistical analyses were performed with SPSS, version 20 software (IBM Corp., Armonk, NY, USA). Correlation analyses included Pearson product-moment correlations for two continuous variables and Spearman’s rank correlations when at least one variable was ordinal. COVID-19-related worry was classified retrospectively, by forming value groups. In this analysis, the area under receiver operating characteristic curve (AUC) was used to determine the cutoff value for the level of COVID-19-related worry capable of distinguishing smoking status groups. For association analyses, binary logistic regressions were performed to calculate odds ratios (OR) with their respective 95% confidence intervals (CI). Odds ratios were reported when data were compared cross-sectionally. Adjusted OR (aOR) were calculated by adjusting for sociodemographic and clinical characteristics including: sex (male/female), age, race/ethnicity, income, education, housing (house/apartment or homeless), employment status, and presence of diabetes, hypertension, heart disease, and HIV. Additionally, tests were performed for multiple interactions on the OR scale-logistic regression with a cross-product.

## 3. Results

### 3.1. Participant Characteristics

Table 1 displays the sociodemographic and clinical characteristics of the 314 participants who completed the COVID-19 survey, comparing participants with and without mental disorder(s) at baseline. In total, 123 (39.2%) participants reported at least one mental disorder diagnosis. Of these, 40 (32.5%) participants reported at least two concurrent mental disorder diagnoses. The most frequent diagnoses were depression (72.4%), anxiety disorder (56.9%), bipolar disorder (33.3%), and schizophrenia (23.6%). The mean age of participants was 56.7 ± 6.9 years, 48.1% were male, 77.3% identified as Black non-Hispanic, and 15.3% as Hispanic. Most participants earned below $15,000 annually per household (73.0%). Two-thirds received a high-school education or less (67.2%) and more than half of the participants were unemployed or could not work due to disability (54.1%), with a more significant percentage in the group of participants with mental disorders (64.2% vs. 47.6%, *p* = 0.003). The mean body mass index (BMI) of participants was 30.3 ± 7.0 kg/m^2^; 46.5% were obese, 55% were hypertensive, 18.8% were diabetic, and 20.1% had heart disease. The majority of participants were living with HIV (58.6%), were virally suppressed (HIV RNA < 200 copies/mL), and had a mean CD4+ cell count of 660 ± 356 cells/µL. Comparing substance use between participants with and without mental disorders, we found that 58.0 % vs. 40.8% were smokers (*p* = 0.002). There were no statistically significant differences in the use of alcohol, cocaine, or cannabis. These characteristics closely resemble those of the overall MASH cohort.

### 3.2. COVID-19-Related Stress and Mental Disorders

COVID-19-related worry scores above the cutoff of ≥9 were associated with current cigarette smoking, with significant predictive value for current smokers compared to non-smokers in participants with mental disorder(s) (all participants: AUC = 0.563, 95% CI 0.50–0.62, *p* = 0.054; participants with mental disorder(s): AUC = 0.682, 95% CI 0.58–0.77, *p* = 0.001; and participants without mental disorder(s): AUC = 0.476, 95% CI 0.39–0.55, *p* = 0.576).

As shown in Table 2, 44.6% (*n* = 140) reported high levels of COVID-19-related stress. Participants with mental disorder(s) were more likely to have higher levels of COVID-19-related stress (8.59 [5.0–10.0] vs. 7.65 [5.0–10.0]; *p* = 0.026) and, after adjustment for confounders, higher odds of high levels of worry (aOR = 1.65, 95% CI: 1.01–2.71; *p* = 0.045).

### 3.3. COVID-19 Infection-Related Symptoms

The large majority of participants did not report COVID-19 infection-related symptoms (86.3%). Only 5.4% of participants tested positive for COVID-19. The main symptoms reported were muscle aches (myalgia) (4.1%), shortness of breath (dyspnea) (3.2%), runny nose (rhinorrhea) (2.2%), cough (new onset or worsening of chronic cough) (2.2%), headache (1.9%), diarrhea (more than or equal to three loose/looser than normal stools in a 24 h period) (1.6%), abdominal pain (1.0%), sore throat (0.6%), sudden loss of smell (0.6%), nausea or vomiting (0.3%), and sudden loss of taste (0.3%). Only two participants reported hospitalizations due to COVID-19 (0.6%).

### 3.4. Smoking before and during the Pandemic

Table 3 shows that approximately half of all participants smoked cigarettes; 52.5% before the pandemic and 47.8% during the pandemic (McNemar’s χ^2^ = 5.297, *p* = 0.021). Of those who smoked before the pandemic (52.5%, *n* = 165), 139/165 (84.2%) smoked persistently, and only 26/165 (15.8%) quit smoking during the pandemic. Among nonsmokers before the pandemic (*n* = 138), 11/138 (8.0%) relapsed during the pandemic. The number of smoked cigarettes was greater before the pandemic [median: 6.8, Q1–Q3 = 3–10)] than during the pandemic [(median 6.4, Q1–Q3 = 3–10) (*p* = 0.027, Wilcoxon test)].

The relationship between the presence of mental disorder(s) and smoking habits before and during the pandemic was evaluated with multiple logistic regressions, adjusting for covariates (age, sex, race/ethnicity, income, education, housing, employment, HIV status, alcohol misuse, cocaine use, and cannabis use). Participants with mental disorder(s), compared to those without, were twice as likely to smoke cigarettes both before (aOR = 2.02, 95% CI: 1.21–3.37, *p* = 0.007) and during (aOR = 2.10, 95% CI: 1.24–3.56, *p* = 0.006) the pandemic (Table 3).

### 3.5. COVID-19-Related Worry as a Measure of Stress, and Smoking

In Table 4, we further examined the effect of COVID-19-related worry scores on smoking status during the COVID-19 pandemic, stratified by the presence of a mental disorder. This effect was only significant among participants with mental disorder(s) but not in those without (Table 4).

In Table 5, we assessed the impact of mental disorder(s) and COVID-19-related worry scores as a measure of stress on the frequency of cigarette smoking (days per month and cigarettes per day) during the pandemic. Overall, participants with mental disorder(s) smoked on more days per month (14.9 [0, 30] vs. 5.3 [0, 30]; *p* = 0.002) and tended to smoke more cigarettes per day than those without (7.4 [0–10] vs. 5.5 [0–15]; *p* = 0.053). Moreover, participants with mental disorder(s) who also experienced high levels of COVID-19-related worry smoked more days per month (20.1 [0–30] days) than those with lower levels of worry scores (9.2 [0–30] days, *p* = 0.021), as well as those with high levels of worry scores but no mental disorder(s) (2.6 [0–30] days, *p* < 0.001). Furthermore, among participants with mental disorder(s), those with high levels of COVID-19-related worry smoked 2.8 ± 10.8 more days per month than those with lower levels of COVID-19-related worry, who smoked 1.9 ± 12.2 fewer days per month than those without a mental disorder (*p* = 0.040). This effect was not seen among participants without a mental disorder (Table 5).

### 3.6. COVID-19-Related Worry, as a Measure of Stress, and COVID-19 Symptoms

We did not find a statistically significant relationship between the intensity of COVID-19-related worry and COVID-19 infection symptoms. The mean COVID-19-related worry scores were not different between those who reported COVID-19 infection symptoms and those who did not [(6.9 + 3.3) vs. (7.7 + 2.7); *p* = 0.11) or those who got tested for COVID-19 compared to those who did not [(7.6 + 3.0) vs. (7.0 + 3.2,); *p* = 0.568), or those who tested positive for COVID-19 compared to those who did not [(7.0 + 4.2) vs. (7.4 + 3.2), *p* = 0.904).

## 4. Discussion

This study investigated the relationship between cigarette smoking and mental disorder(s) before (December 2019) and during the COVID-19 pandemic (July/August 2020) and whether it was influenced by COVID-19-related stress among a vulnerable and disadvantaged population, who already face a high prevalence of smoking-related morbidity and mortality [8,17,18].

Our results show that overall cigarette smoking was related to reporting a mental disorder before and during the pandemic. Those who reported having one or more mental disorder(s) were about twice as likely to smoke before the pandemic and continue smoking during the pandemic compared to those without a mental disorder. Although overall cigarette smoking decreased during the pandemic, there was a higher prevalence of cigarette smoking and increased smoking frequency among participants with mental disorder(s) than those without during the pandemic.

Moreover, the highest risk of smoking was seen among participants with mental disorder(s) who also had high levels of COVID-19-related stress. Our study’s population represents a well-characterized, underserved cohort of Black and Hispanic adults known to have socioeconomic disadvantages (e.g., high rates of poverty and low education), as well as behaviors (misuse of alcohol and other substances) and health conditions associated with poor COVID-19 outcomes, such as diabetes and hypertension. Nearly 40% of participants reported a mental disorder before the pandemic, and approximately half of the participants smoked cigarettes. Due to concerns of increased risk of SARS-CoV-2 transmission and poorer COVID-19-related outcomes among smokers, smoking habits in this population might have substantial public health implications.

In this analysis, MASH cohort participants with mental disorder(s) were more likely to smoke, both before and during the pandemic, compared to those without a mental disorder. Our findings agree with previous studies, showing that smoking is highly prevalent among adults with mental disorder(s); they are less likely to quit smoking than adults without a mental disorder [21,22]. As hypothesized, our study revealed that stress related to COVID-19 exacerbated the risk of smoking among those with a preexisting mental disorder. Overall, participants with mental disorder(s) and high levels of COVID-19-related worry scores were five times as likely to smoke persistently than those without either a mental disorder or high levels of worry.

Moreover, smokers with a reported mental disorder and higher COVID-19-related worry scores, as a parameter of stress, smoked almost 20 days per month during the pandemic, more than twice the rate among those with a mental disorder and low stress scores, and more than seven times the rate among those with high stress scores but no reported mental disorder. These findings are consistent with previous studies that have found positive correlations between mental health symptoms and cigarette use, with the most frequent and persistent smoking behaviors found among patients with a diagnosis of a mental disorder [10,11].

The psychosocial impact of the pandemic is plausibly explained by factors such as confinement, loneliness, fear of stigma and discrimination, and fear of the disease itself [23]. This survey took place early in the pandemic when no vaccines were available.

Notably, the COVID-19 pandemic has led to increased concerns for those with pre-existing mental disorders. For example, Costa et al. found that individuals with anxiety disorder, major depression, or bipolar disorder reported coping poorly during the pandemic, and expressed concerns about the potential effects of the pandemic on their mental health, including worsening their illness, preventing treatment, and forestalling prescription refills [16]. These same concerns may have repercussions related to smoking, since stress, fear, and boredom can be triggers for smoking [10,11]. As such, COVID-19-related stress could lead smokers to smoke more frequently as a coping mechanism [10]. Indeed, Patwardhan et al. reported an increased risk of smoking relapse and more frequent smoking among smokers in the U.K. due to the unavailability of programs and support mechanisms during the pandemic [11]. In addition, cigarette smoking can increase the likelihood of relapse among people recovering from substance use disorders [24]. The National Institute on Drug Abuse has recommended that helping patients quit smoking and remain abstinent may improve their chances for sustained recovery from use of other substances [24].

At the time of writing, the relationship between smoking and COVID-19 remains controversial. The World Health Organization has stated that the relationship between smoking and COVID-19 warrants attention since multiple reports suggest that smoking increases the risk of infection and adverse COVID-19 disease outcomes [6,25]. Additionally, it is widely agreed that smoking cessation has enormous health benefits and could reduce the risk of comorbidities associated with COVID-19 disease severity including death [25,26,27]. Our results reaffirm the relationship between mental disorder(s) and cigarette smoking and suggest that acute psychological factors, such as COVID-19-related stress, may exacerbate smoking habits. Thus, comprehensive treatment and support for mental health should be part of public health efforts to mitigate the impact of the COVID-19 pandemic. This may be particularly important for members of disadvantaged groups, such as the MASH cohort, who are predisposed to substance use disorders, the most common being nicotine dependence. Proactive measures to provide mental health care and tobacco use cessation support during times of crisis may be beneficial both in the short-term and long-term in the aftermath of the pandemic. Our findings underscore the value of examining the impact of mental health on substance use and vice versa.

## 5. Strengths and Limitations

Several strengths and limitations of this study should be acknowledged. This study was limited by its use of self-reported data. Nonetheless, we used validated and widely used instruments. The study’s observational nature also prevents us from establishing causality between the presence of mental disorder(s) and smoking. In this study, we evaluated the frequency of cigarette consumption; future studies may consider measures of nicotine dependence.

The study is strengthened by its use of longitudinal data on mental disorders reported prior to the pandemic, which were compared with smoking behaviors during the pandemic, establishing a temporal association. While our findings may not be generalized to the entire population, the MASH cohort is primarily comprised of low-income, Black non-Hispanic and Hispanic, middle-aged adults living with and without HIV. Therefore, this study provides insights into the problems facing underserved U.S. minorities.

## 6. Conclusions

The number of participants who smoked decreased during the pandemic, but those with mental disorder(s) and higher levels of COVID-19-related worry, as a parameter of higher stress, were more likely to smoke and at a higher frequency than their peers with less COVID-19-related stress. The severity of COVID-19-related stress in those with mental disorder(s) significantly increased the risk of smoking in these individuals.

## Figures and Tables

**Table 1:** Socioeconomic and clinical baseline characteristics of participants with and without pre-existing mental disorders.

Clinical Characteristic	Total (*n* = 314)	Mental Disorder (*n* = 123)	No Mental Disorder (*n* = 191)	*p*
	% or Mean ± SD	% or Mean ± SD	% or Mean ± SD	
**Age**, years	56.7 ± 6.9	55.9 ± 6.3	57.3 ± 7.2	0.081 *
**Sex**, male	48.1%	43.1%	51.3%	0.095 **
**Race/ethnicity**				0.058 ‡
Black non-Hispanic	77.3%	70.5%	81.7%	
White non-Hispanic	7.4%	10.7%	5.2%	
Hispanic	15.3%	18.8%	13.1%	
**Income**				0.614 ‡
$15,000 or less	73.0%	76.1%	70.8%	
$15,000−$30,000	21.2%	18.8%	22.9%	
$30,000 or more	5.8%	5.1%	6.3%	
**Education**				0.432 ‡
Less than high school	36.9%	36.6%	37.2%	
High school or GED	30.3%	26.8%	32.4%	
More than high school	32.8%	36.6%	30.4%	
**Housing**				
House/apartment	95.2%	92.7%	96.9%	0.414 **
Homeless	4.8%	7.3%	3.1%	
**Not working**	54.1%	64.2%	47.6%	0.003 **
**Morbidities**				
BMI	30.3 ± 7.0	30.6 ± 7.1	30.1 ± 6.9	0.472 *
Obesity	46.5%	49.6%	44.5%	0.418 **
Hypertension	55.0%	57.7%	55.0%	0.359 **
Diabetes	18.8%	17.9%	19.4%	0.431 **
Heart disease	20.1%	19.4%	21.1%	0.404 **
HIV	58.6%	56.9%	59.7%	0.355 **
**Substance use**				
Cigarette smoking	47.8%	58.5%	40.8%	0.002 **
Alcohol misuse	34.4%	33.3%	35.1%	0.423 **
Cocaine	12.4%	12.2%	12.6%	0.535 **
Cannabis	23.9%	27.6%	21.5%	0.132 **

* Student’s *t*-test; ** Chi-square test; ‡ Likelihood ratio Chi-square test; Bold indicates *p*-value < 0.05.

**Table 2:** Comparison of COVID-19-related worry scores as a measure of stress between participants with and without existing mental disorders.

	Stress (COVID-19-Related Worry)			
	Worry Scale ^1^	High Worry ^1^	OR ^2^	aOR ^2,3^
Total (*n* = 314)	8.03 (5.0–10.0)	44.6%		
With mental disorder (*n* = 123)	8.59 (5.0–10.0)	51.2%	1.55 (0.98–2.45)	1.65 (1.01–2.71)
Without mental disorder (*n* = 191)	7.65 (5.0–10.0)	40.3%		
*p*	**0.026**		0.058	**0.045**

Abbreviations: aOR, adjusted odds ratio; OR, odds ratio; Bold indicates *p*-value < 0.05. Data are presented as median (with first and third quartiles) using non-parametric Mann-Whitney U test for comparison. COVID-19-related worry was measured on a scale of 1 to 10, with scores of ≥9 indicating high levels of worry. ^1^ Logistic regressions were performed using participants without a mental disorder as the reference group. ^2^ Estimates are adjusted for age, sex, race/ethnicity, income, education, housing, employment, HIV status, cigarette smoking, alcohol misuse, cocaine use, and cannabis use ^3^.

**Table 3:** Smoking behaviors before and during the COVID-19 pandemic among participants with and without mental disorders.

Smokers		Mental Disorders				
	Total	Yes	No	aOR ^4,5^	95% CI	*p*
Before pandemic ^1^	52.5%	61.8%	46.6%	1.85	1.14–2.85	**0.012**
During pandemic ^1^	47.8%	58.5%	40.8%	2.00	1.23–3.24	**0.005**
Persistent smokers ^2^	44.3%	55.3%	37.2%	2.04	1.26–3.30	**0.004**
Quit smoking ^3^	15.8%	10.5%	20.2%	0.51	0.20–1.31	0.165

Abbreviations: aOR, adjusted odds ratio; CI, confidence interval; Bold indicates *p*-value < 0.05. Smokers: participants who smoked on at least one of the 30 days prior to the interview, surveyed separately before and during the pandemic. ^1^ Persistent smokers: participants who smoked both before and during the pandemic. ^2^ Quit smoking: participants who were classified as a smoker pre-pandemic but as a non-smoker during the pandemic. ^3^ Logistic regression was performed using participants without a mental disorder as the reference group to estimate the odds of smoking given the presence of a mental disorder. ^4^ Estimates are adjusted for sociodemographic and clinical characteristics (age, sex, race/ethnicity, income, education, housing, employment, diabetes, hypertension, heart disease, and HIV status) ^5^.

**Table 4:** Effect of COVID-19-related worry scores, as a measure of stress, on smoking status during the COVID-19 pandemic stratified by the presence of a mental disorder.

Mental Disorders	COVID-19 Worry	Number	Smoking Behavior				
			Yes	No	aOR ^1,2^	95% CI	*p*
			**Smoking during the pandemic ^3^**				
No	Low	114	44.7%	55.3%	Reference		-
	High	77	35.1%	64.9%	0.78	0.39–1.63	0.522
Yes	Low	60	46.7%	53.3%	Reference		
	High	63	69.8%	30.2%	3.36	1.45–7.75	**0.004**
			**Persistent smokers ^4^**				
No	Low	56	46.5%	53.5%	Reference		-
	High	33	36.9%	63.1%	0.97	0.30–3.45	0.967
Yes	Low	32	50.0%	50.0%	Reference		
	High	44	71.9%	28.1%	7.76	1.13–53.50	**0.037**

Abbreviations: aOR, adjusted odds ratio; CI, confidence interval; Bold indicates *p*-value < 0.05. Binary logistic regression analyses were performed for the odds of smoking among those with vs. without high levels of COVID-19-related worry. ^1^ Estimates are adjusted for age, sex, race/ethnicity, income, education, housing, employment, HIV status, alcohol misuse, cocaine use, and cannabis use. ^2^ Among smokers in pre-pandemic timepoint only (*n* = 165). ^3^ Among smokers who smoked both before and during the pandemic (*n* = 139) ^4^.

**Table 5:** Influence of COVID-19-related worry scores, as a measure of stress, on smoking frequency during the pandemic ^1^.

		**Smoke Days/Month**				**Cigarettes/Day**		
		**Mental Disorder**				**Mental Disorder**		
**Population**	**All**	**Yes**	**No**	***p* ^2^**	**All**	**Yes**	**No**	***p* ^2^**
**All**	9.0 (0–30)	14.9 (0–30)	5.3 (0–30)	0.002	6.4 (3–10)	7.4 (0–10)	5.5 (0–15)	0.053
**Stress** **(COVID-19-related worry)**								
**Low**	7.8 (0–30)	9.2 (0–30)	7.2 (0–30)	0.637	6.3 (3–10)	8.2 (4–15)	5.6 (3–10)	0.115
**High**	10.5 (0–30)	20.1 (0–30)	2.6 (0–30)	**<0.001**	6.5 (3–10)	7.0 (3.7–11.2)	5.7 (3–10)	0.219
***p* ^3^**	0.314	**0.021**	0.248		0.994	0.589	0.771	

Bold indicates *p*-value < 0.05. Data are presented as median and the first and third quartiles using non-parametric Mann-Whitney U test for comparison. COVID-19-related worry was measured with a scale of 1 to 10, with scores of ≥9 indicating high levels of worry. ^1^ Comparison between participants with and without a mental disorder. ^2^ Comparison between participants with high vs. low COVID-19-related worry ^3^.

## Data Availability

Data will be made available upon request (baumm@fiu.edu).
